# Correlation between the mutation rates for *ampC* derepression and species or *ampC* genotypes in *Enterobacter cloacae* complex

**DOI:** 10.1186/s12866-025-04310-y

**Published:** 2025-09-24

**Authors:** Lianyan Xie, Yanrong Wu, Qiulan Huang, Jingyong Sun

**Affiliations:** 1 Department of Laboratory Medicine, Ruijin Hospital, Shanghai Jiao Tong University School of Medicine, Shanghai, China; 2 School of Basic Medical Sciences, Tsinghua University, Beijing, China; 3 Department of Clinical Laboratory, Nanxiang Hospital Jiading District Shanghai, Shanghai, China

**Keywords:** *Enterobacter cloacae* complex, AmpC β-lactamase, Mutation rate, *AmpC* genotype, Species

## Abstract

**Background:**

In this study, we investigated the correlation between the mutation rates for *ampC* derepression and species or *ampC* genotypes in *Enterobacter cloacae* complex (ECC) susceptible to ceftriaxone. Non-duplicate ceftriaxone-sensitive ECC isolates (90) were obtained from September 2021 to January 2023 at Ruijin Hospital, Shanghai. *hsp60* genotyping and PCR were used for species and *ampC* identification, respectively. Thirteen strains with the negative *ampC* amplification results were sequenced, and the mutation rates for *ampC* derepression were determined by performing Luria–Delbrück fluctuation analyses.

**Results:**

Among these isolates, *E. hormaechei* was the most prevalent (54.44%), followed by *E. roggenkampii* (12.22%), *E. cloacae* (11.11%), *E. bugandensis* (10.00%), *E. asburiae* (4.44%), and *E. kobei* (4.44%). For *E. ludwigii*, *E. mori*, and *E. sichuanensis*, only a single strain was identified. There were 72 strains with *bla*_ACT_, 13 with *bla*_MIR_, and five with *bla*_CMH_. The *ampC*-derepressed mutation rate was (2.25 ± 1.81) × 10^−8^ for *E. asburiae*, (3.21 ± 2.96) × 10^−8^ for *E. bugandensis*, (6.06 ± 11.95) × 10^−8^ for *E. cloacae*, (1.12 ± 3.44) × 10^−7^ for *E. hormaechei*, (7.76 ± 11.41) × 10^−8^ for *E. kobei*, (3.99 ± 9.65) × 10^−8^ for *E. roggenkampii*, 5.87 × 10^−8^ for *E. ludwigii*, 1.12 × 10^−7^ for *E. mori*, and 2.17 × 10^−7^ for *E. sichuanensis.* The mutation rate was (8.94 ± 28.61) × 10^−8^ for *bla*_ACT_, (2.62 ± 2.16) × 10^−8^ for *bla*_CMH_, and (8.10 ± 13.84) × 10^−8^ for *bla*_MIR_.

**Conclusions:**

ECC was found to have high mutation rate−inducible AmpC production with no species or *ampC* genotype differences. This highlights an important clinical concern, i.e., the high risk of treatment failure with third-generation cephalosporins in individuals with inducible AmpC-containing ECC.

**Supplementary Information:**

The online version contains supplementary material available at 10.1186/s12866-025-04310-y.

## Background

*Enterobacter cloacae* complex (ECC) comprises opportunistic infectious pathogens that have become a source of concern in hospital environments in recent decades [1]. ECC pathogens cause numerous infections, including bacteraemia, pneumonia, urinary tract infections, biliary tract infections, brain abscesses, and endocarditis [2–4]. ECC includes more than 20 species based on physiological and genetic characteristics [1]. In clinical laboratories, routine identification of ECC species is performed using matrix-assisted laser desorption/ionisation, time-of-flight (MALDI-TOF) mass spectrometry (MS). Although this method can effectively identify ECC, it does not differentiate between subspecies [5]. Heat shock protein 60 (*hsp60*) typing is a highly efficient method for ECC species-level identification based on the analysis of *hsp60* gene segment sequences. This method can be used to group ECC members into 12 genetic clusters (I–XII) and one unstable sequence cluster (XIII) [6]. Recently, whole-genome sequencing has revealed 22 phylogenetic clades (A–V) within ECC, further illustrating its complex taxonomy [7].

ECC is primarily associated with chromosome AmpC enzyme production which hydrolyse penicillins, cephalosporins and monobactams [8]. And the use of third-generation cephalosporins (3GCs), such as ceftriaxone or ceftazidime, to treat infections caused by these sensitive isolates is often unsuccessful owing to the derepression of AmpC β-lactamases [9, 10]. Multiplex PCR has been used to identify family-specific *ampC* genes responsible for AmpC β-lactamase expression in organisms, and using this approach, six different groups have been identified based on percentage similarities, namely, ACC (origin *H. alvei*); FOX, MOX, and DHA (origin *M. morganii*); CIT (origin *C. freundii*); and EBC (origin *E. cloacae*) [11].

However, differences among the different clinical isolates of ECC with respect to the rates of *ampC* derepression mutation remain, and the correlation of species and *ampC* genotype with the mutation rates for *ampC* derepression in ECC has yet to be determined. Thus, in this study, we aimed to investigate the correlation of species and *ampC* genotype with the mutation rates for *ampC* derepression in ECC to determine if single species or *ampC* genotypes were associated with different mutation rate for *ampC* derepression. Our findings are anticipated to contribute to estimating the risk of treatment failure when using 3GCs.

## Materials and methods

### Strain source and antimicrobial susceptibility testing

A total of 90 non-duplicate ECC strains sensitive to ceftriaxone were isolated from Ruijin Hospital, Shanghai, China, between September 2021 and January 2023. The strains were identified using the VITEK MS system (bioMérieux, Marcy-l’Étoile, France). Antimicrobial susceptibility testing of ceftriaxone was performed using disc diffusion assays according to the Clinical and Laboratory Standards Institute (CLSI) M100-S33 [12]. *Escherichia coli* ATCC 25,922 was used as a quality control.

### Species identification

Species identification was performed based on *hsp60* gene analysis using PCR (Table 1) [13]. The PCR amplification programme was as follows: an initial denaturation at 95℃ for 10 min; 30 cycles at 95℃ for 30 s, 57.5℃ for 30 s, and 72℃ for 1 min; and a final extension step at 72℃ for 10 min. The sequences thus obtained were compared with those of 13 subspecies within the ECC, which were retrieved from the GenBank database (http://www.ncbi.nlm.nih.gov).

**Table 1:** The primers used in this study

Gene	Primer	Sequence (5′−3′)	Expected amplicon size (bp)	Reference
*hsp60*	hsp60-F	GGTAGAAGAAGGCGTGGTTGC	341	[13]
	hsp60-R	ATGCATTCGGTGGTGATCATCAG		
MOX	MOXMF	GCTGCTCAAGGAGCACAGGAT	520	[11]
	MOXMR	CACATTGACATAGGTGTGGTGC		
CIT	CITMF	TGGCCAGAACTGACAGGCAAA	462	[11]
	CITMR	TTTCTCCTGAACGTGGCTGGC		
DHA	DHAMF	AACTTTCACAGGTGTGCTGGGT	405	[11]
	DHAMR	CCGTACGCATACTGGCTTTGC		
ACC	ACCMF	AACAGCCTCAGCAGCCGGTTA	346	[11]
	ACCMR	TTCGCCGCAATCATCCCTAGC		
FOX	FOXMF	AACATGGGGTATCAGGGAGATG	302	[11]
	FOXMR	CAAAGCGCGTAACCGGATTGG		
EBC	EBCMF	TCGGTAAAGCCGATGTTGCGG	190	[11]
	EBCMR	CTTCCACTGCGGCTGCCAGTT		

### AmpC genotyping

PCR was performed using specific primers to detect the AmpC gene (*bla*_ACC_, *bla*_DHA_, *bla*_EBC_, *bla*_FOX_, *bla*_MOX,_ and *bla*_CIT_), as previously described (Table 1) [11]. The products were separated by electrophoresis using 1% agarose gels with 0.5× TBE buffer, stained with 4SGelred, and visualised under ultraviolet illumination. The Basic Local Alignment Search Tool (BLAST) at the National Center for Biotechnology Information website was used for sequence analysis.

### DNA preparation, sequencing, and analysis

Thirteen strains with the negative *ampC* amplification results were sent for genome sequencing. Genomic DNA was extracted using a Wizard^®^ Genomic DNA Purification Kit (Promega, Madison, WI, USA) according to the manufacturer’s protocol. DNA libraries were compiled using a NEXTflexTM Rapid DNA-Seq Kit (Bioo Scientific, Austin, TX, USA), and sequencing was conducted using 2 × 150 bp alignment reads using an Illumina NovaSeq 6000 sequencer. Raw reads obtained after sequencing were filtered using fastp software (version 0.19.6) [14], followed by assembly using SOPA de novo version 2.04 [15]. All analyses were performed using the publicly available online Majorbio Cloud Platform from Shanghai Majorbio Bio-pharm Technology Co., Ltd. Precise species identification, dependent on the average nucleotide identity, was performed using JSpeciesWS based on BLAST [16]. The sequences for the 13 strains were submitted to GenBank under accession Nos. JAYFUO000000000–JAYFUX000000000 and JBDPIK000000000-JBDPIM000000000.

### Mutation rates

To calculate mutation rates, we performed Luria–Delbrück fluctuation analyses, as outlined by Rosche and Foster [17]. Fluctuation tests involved inoculating a few cells into a larger number of parallel cultures, each of which was then plated on a selective medium to determine the number of mutants. Total cell counts were also determined using dilutions in non-selective culture medium. The mutation rate was determined based on the distribution of the number of mutants in the culture. For each of the assessed strains, 4 mL of a 10^5^ CFU/mL bacterial suspension in Mueller–Hinton (MH) broth was incubated overnight at 37℃. Two cultures (100 µL of bacterial suspension cultured on the previous day) were used to determine bacterial counts by using serial dilutions to inoculate non-selective MH agar, which were then incubated overnight at 37℃. An additional 10 cultures (100 µL of bacterial suspension cultured the previous day) were used to inoculate MH agar containing ceftriaxone (8 mg/L) to determine the number of mutants. Based on the distributions of the mutant numbers in the cultures, we estimated the probable number of mutants using the MSS maximum likelihood method (https://lianglab.brocku.ca/FALCOR/) [17, 18]. Mutation rates for each cell generation were calculated based on the number of mutations and bacterial counts with In(2) correction, as suggested by Armitage [19].

### Statistical analysis

The data analysis function of GraphPad Prism 8.0.2 was used for statistical analysis of the correlation between the high expression mutation rate of chromosomally induced *ampC* disinhibition and the species or genotype of all ECC clinical isolates. Welch’s analysis of variance (ANOVA) test (*n* > 1) was performed for statistical analysis and Games-Howell’s multiple comparisons test was performed for compare groups pairwise as appropriate, with a P-value < 0.05 being considered statistically significant.

## Results

### Strain source and identification

In this study, we identified 90 ECC strains that were primarily isolated from sputum (38/90, 42.22%), secretions (17/90, 18.89%), and urine (10/90, 11.11%). Among these 90 strains, we identified the nine species, namely, *E. asburiae*, *E. bugandensis*, *E. cloacae*, *E. hormaechei*, *E. kobei*, *E. ludwigii*, *E. mori*, *E. roggenkampii*, and *E. sichuanensis*, among which *E. hormaechei* showed the highest prevalence (49/90, 54.44%), followed by *E. roggenkampii* (11/90, 12.22%), *E. cloacae* (10/90, 11.11%), *E. bugandensis* (9/90, 10.00%), *E. asburiae* (4/90, 4.44%), and *E. kobei* (4/90, 4.44%). In the case of *E. ludwigii*, *E. mori*, and *E. sichuanensis*, only single strains were identified.

### AmpC genotyping

Based on *ampC* genotyping, we identified 72 strains (72/90, 80.00%) as *bla*_ACT_, 13 (13/90, 14.44%) as *bla*_MIR_, and five (5/90, 5.56%) as *bla*_CMH_. ACT variants were identified in *E. hormaechei* (49/72, 68.05%), *E. bugandensis* (9/72, 12.50%), *E. asburiae* (4/72, 5.56%), *E. cloacae* (4/72, 5.56%), *E. kobei* (4/72, 5.56%), *E. ludwigii* (1/72, 1.39%), and *E. mori* (1/72, 1.39%), and MIR variants were identified in *E. roggenkampii* (11/13, 84.62%), *E. cloacae* (1/13, 7.69%), and *E. sichuanensis* (1/13, 7.69%). CMH variants were detected only in *E. cloacae* (5/5, 100%).

### Mutation rates

**Fig. 1:**
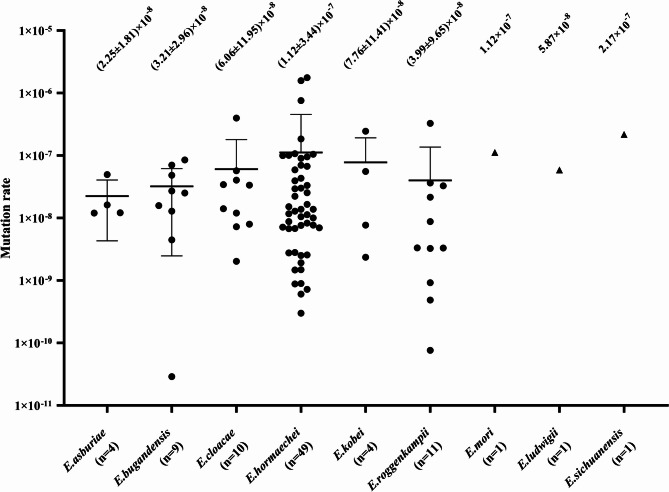
Species mutation rates. Mutation rates were determined for each strain using black dots. Species with *n* = 1 were excluded from statistical analysis, and their mutation rates were represented by black triangles. Results are grouped according to species. The horizontal black lines in each column indicate the species mean mutation rate. The mean mutation rate is displayed as mean ± standard deviation (SD)

**Fig. 2:**
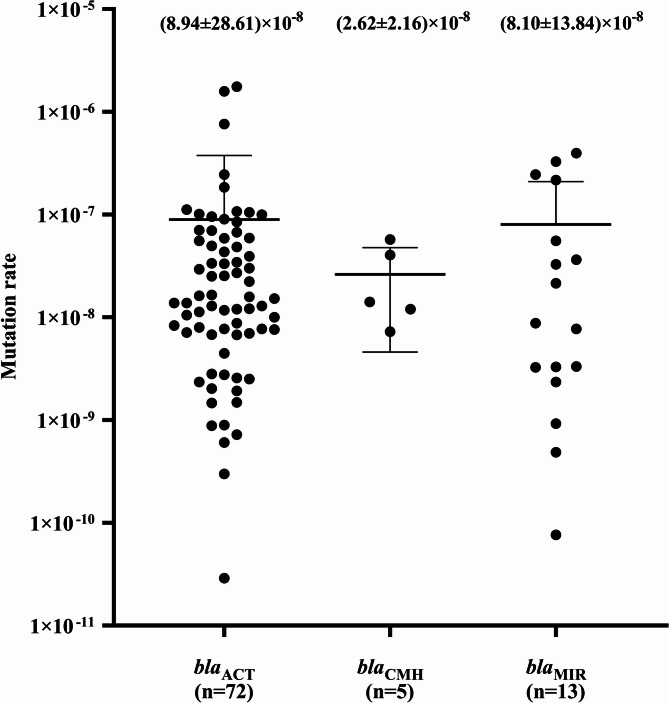
*ampC* genotype mutation rates. Mutation rates were determined for each strain using black dots. Results are grouped according to *ampC* genotypes. The horizontal black lines in each column indicate *ampC* genotype mean mutation rate. The mean mutation rate is displayed as mean ± standard deviation (SD)

As shown in Fig. 1, the *ampC*-derepressed mutants have mutation rates of (2.25 ± 1.81) × 10^−8^ for *E. asburiae*, (3.21 ± 2.96) × 10^−8^ for *E. bugandensis*, (6.06 ± 11.95) × 10^−8^ for *E. cloacae*, (1.12 ± 3.44) × 10^−7^ for *E. hormaechei*, (7.76 ± 11.41) × 10^−8^ for *E. kobei*, (3.99 ± 9.65) × 10^−8^ for *E. roggenkampii*, 5.87 × 10^−8^ for *E. ludwigii*, 1.12 × 10^−7^ for *E. mori*, and 2.17 × 10^−7^ for *E. sichuanensis*. The mean mutation rate of ECC species was (8.46 ± 26.10) × 10^−8^, among which the mutation rate of *E. asburiae* was five-fold lower than that of *E. hormaechei*. However, we detected no statistically significant differences (*P* > 0.05) in the mean mutation rates among species in ECC. The P-values for pairwise comparisons are between 0.47 and 0.97. Given that we identified only single strain of *E. Ludwigii*,* E. mori*, and *E. sichuanensis*, these were excluded from the statistical analysis. As shown in Fig. 2, the mutation rates obtained for the *ampC* genotypes were (8.94 ± 28.61) × 10^−8^ for *bla*_ACT_, (2.62 ± 2.16) × 10^−8^ for *bla*_CMH_, and (8.10 ± 13.84) × 10^−8^ for *bla*_MIR_. Although the rates were found to be similar for *bla*_ACT_ and *bla*_MIR_, they were three-fold higher than those detected for *bla*_CMH_. However, the P-value of 0.111 obtained based on Welch’s ANOVA and the P-value between 0.18 and 0.98 obtained for pairwise comparisons indicated that there were no statistically significant differences in the mean mutation rates among different *ampC* genotypes in ECC.

## Discussion

Over the past decade, ECC has become a widespread group of pathogens capable of causing a range of disorders, including pneumonia, urinary tract infections, and septicaemia [1, 20]. The strains assessed in this study were predominantly isolated from sputum samples, followed by secretions and urine samples. Given their constitutive low-level expression of AmpC β-lactamase, bacteria in ECC have intrinsic resistance to ampicillin, amoxicillin, amoxicillin-clavulanate, and early generation cephalosporins [9]. AmpC regulation is complicated, involving *ampR*, *ampD*, and *ampG*, all of which are related to peptidoglycan recycling [8]. In normal conditions, degradation products are transported from the cell wall to cytoplasm *via* AmpG, where they are cleaved by AmpD. Next, they bind to AmpR, giving rise to a conformation that inhibits the transcription of *ampC*. Exposure to β-lactams leads to increased accumulation of some peptides, and AmpD is unable to efficiently process the remaining fragments. AmpR’s activity is impaired when it is bound by these remaining fragments, which can constitutively induce high expression of *ampC* [21]. This phenomenon is called derepression. The highly homologous AmpR-AmpC system exists in *Pseudomonas aeruginosa* and *Enterobacteriaceae*, but the reasons for excessive AmpC production differ slightly. For instance, the mutation-based inactivation of *dacB* (encoding penicillin binding protein 4) may be the most common cause in *P. aeruginosa*, whereas in *Enterobacteriaceae* its impact seems more limited and variable depending on the species. There are more descriptions of AmpC hyperproduction—to various extents—associated with different mutations in *ampD* in *Enterobacteriaceae* [22]. Additionally, other genes, such as *nagZ* (encoding β-N-acetylglucosaminidase), are suggested to play important roles in the regulation mechanism leading to *ampC*-mediated high-level β-lactam resistance in ECC [23]. Numerous reported cases of resistance developing during the clinical application of 3GC treatment have been reported, thereby leading to treatment failure, particularly when the pathogen is present in the blood [24, 25]. Among the populations, the mean mutation rate for *ampC*-derepressed ECC was high (3 × 10^−8^) when compared to that of *Serratia* spp., *Providencia* spp., and *Morganella morganii* (approximately 3 × 10^−10^–3 × 10^−11^). Our detailed examination of the mutation rates of the ECC species and the results of fluctuation assays revealed that the mean mutation rate of ECC species is consistent with values reported in previous studies. However, we detected no statistically significant difference among ECC species in terms of mean mutation rates for *ampC* derepression (*n* > 1 fit into the statistical analyses), thereby indicating that all species in this complex are similarly prone to mutation. Among these species, *E. hormaechei* was the most frequently isolated, which mean mutation rate was five-fold higher than that of *E. asburiae*. The findings of previous studies have indicated that *E. hormaechei* may be more virulent than other ECC species because of its high-pathogenicity island [26]. As a consequence of an increasing number of phylogenetic analyses of ECC, the emergence of antibiotic-resistant *E. hormaechei* isolates, including those producing AmpC β-lactamases, ESBLs, and carbapenemases, is more frequently reported. It has been established that *E. hormaechei* plays an important role in virulence and drug resistance; and thus, mutation rates among ECC strains should be further investigated.

A standard numbering scheme of AmpC β-lactamases based on both sequence and structure has been developed by Mack et al. [27], and the diversity of *ampC* genes in *Enterobacter* species and the association between AmpC variants and *Enterobacter* species has previously been investigated by Feng et al. [28], the findings of which indicate that *bla*_ACT_ originated from several different ECCs, including *E. xiangfangensis*, *E. hoffmannii*, *E. asburiae*, *E. ludwigii*, and *E. kobei*, whereas the origins of *bla*_MIR_ and *bla*_CMH_ have been identified as *E. roggenkampii* and *E. cloacae*, respectively, In the present study, we detected three *ampC* genes, *bla*_ACT_, *bla*_CMH_, and *bla*_MIR_, in the ECC, the most common of which was *bla*_ACT_, which was identified in different species, followed by *bla*_MIR_, which was primarily identified in *E. roggenkampii*, and *bla*_CMH_, which originated from *E. cloacae*. Other data has provided evidence to indicate that *bla*_DHA_ is descended from *Morganella morganii ampC*, and *bla*_ACC_ is descended from *Hafnei alvei ampC* [29], whereas *bla*_DHA−1_ and *bla*_ACT−1_ have been detected in *Serratia marcescens* in China [30]. However, whereas the correlation between the *ampC* genotype and mutation rate may be applicable to other species with *ampC*, in the ECC, we detected similar rates of mutation among *bla*_ACT_, *bla*_MIR_, and *bla*_CMH_. Although the MALDI-TOF MS commercial bacterial identification platform is widely used in clinical microbiology laboratories, it can only identify ECCs at the “complex group” level, and in the present study, we detected no statistically significant difference for species or *ampC* genotypes identification based on mean mutation rates for *ampC* derepression. Nevertheless, the identification of bacterial strains to the “complex group” level is sufficient for clinical doctors to assess medication risks. For ECC and other strains with chromosomally encoded inducible AmpC β-lactamase, CLSI indicates that initially susceptible isolates may become resistant within 3 to 4 days of the initiation of therapy, and therefore repeated analysis of isolates may be warranted [12]. Irrespective of in vitro susceptibility, EUCAST discourages the use of 3GCs for the treatment of severe infections caused by *Enterobacteriaceae* that produce inducible AmpC [31]. For treatment, experts recommend the use of cefepime or carbapenems instead, and ceftazidime/avibactam as an alternative to carbapenems for infections with a moderate to high risk of clinically significant AmpC-producing strains, particularly if it is difficult to identify the source [32–35]. Due to rising resistance, research on alternative methods besides antibiotics is urgently needed. The role of metal-based antimicrobials, such as silver nanoparticles, has recently been explored for their potential to target resistant strains of Mycobacterium tuberculosis, offering an alternative approach to drug resistance suppression in bacterial pathogens [36].

In this study, we comprehensively analysed the mutation rates associated with *ampC* derepression across different species and *ampC* genotypes within the ECC. Our findings demonstrate that the potential for inducible AmpC production-leading to antibiotic resistance-is consistently high across all species and genotypes examined. This underscores the importance of evaluating the risk of treatment failure when using 3GCs to treat patients with ECC harbouring inducible AmpC, even when the isolates initially appear susceptible.

### Limitations

This is a single-centre study, a feature that limits the size, coverage, and diversity of the sample pool. Some ECC species were represented by only one isolate, which makes statistical comparison difficult. In future work, we plan to expand the study by including more medical centres and a broader range of ECC species to improve the robustness of the data. Additionally, we used PCR-based methods to identify *ampC* and *hsp60* genes for species identification, which may introduce some bias in subtyping. A more comprehensive approach, such as whole-genome sequencing of a larger number of isolates, could provide a more accurate classification and meaningful stratifications based on *ampC* variations. We will conduct future studies to address this limitation.

## Supplementary Information

Supplementary Material 1.

## Data Availability

Sequence data that support the findings of this study have been deposited in the NCBI’s Sequence Read Archive under Genome Accession Nos. JAYFUO000000000–JAYFUX000000000 and JBDPIK000000000-JBDPIM000000000.

## Abbreviations

ANI: Average nucleotide identity
ANOVA: Analysis of variance
BLAST: Basic local alignment search tool
ECC: Enterobacter cloacae complex
MH: Mueller–Hinton
MS: Mass spectrometry
PCR: Polymerase chain reaction
